# Clinical and Dermoscopic Determinants of Surgical Decision-Making in Skin Cancer Screening: A Retrospective Analysis

**DOI:** 10.3390/medicina62091655

**Published:** 2026-08-28

**Authors:** Adrian Chwojnicki, Sebastian Makuch, Hanna Adamska, Michalina Świetlicka, Maja Hrynczyszyn, Inga Buława, Paulina Piesch, Jacek Polański, Wojciech Tański

**Affiliations:** 1 Department of Dermato-Venereology, 4th Military Clinical Hospital, 50-981 Wroclaw, Poland; chwoj@wp.pl; 2 Department of Clinical and Experimental Pathology, Wroclaw Medical University, 50-368 Wroclaw, Poland; 3 Faculty of Medicine, Wroclaw University of Science and Technology, 50-370 Wroclaw, Polandwtanski@4wsk.pl (W.T.); 4 Department of Internal and Occupational Diseases, Hypertension and Clinical Oncology, Wroclaw Medical University, 50-556 Wroclaw, Poland; jacek.polanski@umw.edu.pl; 5 Department of Internal Medicine, 4th Military Clinical Hospital, 50-981 Wroclaw, Poland

**Keywords:** melanoma, basal cell carcinoma, dermoscopy, skin cancer screening, atypical nevi, surgical decision, logistic regression, multiple correspondence analysis, screening referral efficiency

## Abstract

*Background and Objectives:* Deciding which pigmented or non-pigmented skin lesions require surgical excision, as opposed to continued observation, is a common challenge in everyday dermatologic practice. Although dermoscopy is well established as a tool that improves melanoma detection accuracy compared with naked-eye examination, real-world evidence on how demographic profile, patient history, and dermoscopic impression jointly influence the surgical decision remains limited. This study examined the clinical and dermoscopic factors associated with a positive surgical decision (PSD) in a large single-centre screening cohort. *Materials and Methods:* This retrospective cross-sectional analysis included 643 consecutive patients (415 female (64.5%), 228 male (35.5%); mean age 51.3 ± 16.8 years) undergoing dermoscopic skin cancer screening. Data recorded included family history of melanoma (FHM), personal history of skin cancer (HSC), childhood sunburn (CS), clinically atypical lesions (CAL), suspicion of basal/squamous cell carcinoma (BCC/SCC), nevus count (NC, 0–4), and the surgical decision (PSD). Free-text dermoscopic descriptions were grouped into seven diagnostic categories. Group comparisons used the Mann–Whitney U, χ^2^, and Fisher’s exact tests; predictors of PSD were characterised using multivariable logistic regression (with Hosmer–Lemeshow and Nagelkerke R^2^ statistics), multiple correspondence analysis (MCA), Gower-distance k-medoids clustering, and receiver operating characteristic (ROC) analysis, including the area under the curve (AUC). Missing NC data (13.2%) were assessed using Little’s test for data missing completely at random (MCAR test). *Results:* A positive surgical decision was recorded in 133/643 patients (20.7%) and was significantly more common in men than women (25.9% vs. 17.9%; *p* = 0.017; corresponding to an odds ratio (OR) of 1.61). In multivariable analysis, suspicion of BCC/SCC (OR = 24.7, 95% confidence interval (CI): 9.26–66.0) and clinically atypical lesions (OR = 5.53, 95% CI 3.50–8.74) were the strongest independent predictors of PSD (Nagelkerke R^2^ = 0.31; Hosmer–Lemeshow *p* = 0.977), far outweighing family or personal history. A significant interaction between childhood sunburn and nevus count (OR = 1.39, 95% CI 1.16–1.66; *p* < 0.001) predicted clinical atypia. Missing NC data were consistent with a completely random pattern (Little’s χ^2^ = 2.80, df = 2, *p* = 0.247). Dermoscopic impression showed strong discriminative value for PSD: “no concern” had the highest negative predictive value (NPV = 0.927; AUC = 0.762), while “atypical lesion” combined high specificity (0.916) and positive predictive value (0.632; AUC = 0.736). On average, 4.8 patients were examined for every surgical referral generated (a screening-to-referral ratio, not a number needed to screen for a histologically confirmed cancer). *Conclusions:* Surgical decision-making in this cohort was driven almost exclusively by the dermoscopic impression—suspected BCC/SCC and clinical atypia—rather than by family or personal oncological history, while male sex was independently associated with a higher surgical rate. These findings support dermoscopy-led triage protocols and highlight the potential value of standardised risk stratification to optimise referral pathways in routine skin cancer screening.

## 1. Introduction

Keratinocyte carcinomas—basal cell carcinoma (BCC) and cutaneous squamous cell carcinoma (cSCC)—together with cutaneous melanoma, represent the majority of the global skin cancers. The Global Burden of Disease modelling indicates that the age-standardised incidence of cSCC increased by 36.1% between 1990 and 2019, with continued growth projected in most regions through 2035 [1]. Melanoma follows a similar trend: GLOBOCAN-based estimates attributed approximately 325,000 new cases and 57,000 deaths to cutaneous melanoma in 2020, with both figures projected to rise substantially—by roughly 50% and 68%, respectively—by 2040 if current patterns persist [2]. Recent studies on mortality suggest that in several high-income countries, melanoma death rates have improved even as new diagnoses keep rising [3] This gap is thought to result partly from increased screening, which detects more slow-growing, subclinical lesions rather than reflecting a genuine increase in life-threatening disease [4]. This growing and increasingly complex clinical demand—driven by population ageing, cumulative ultraviolet exposure, and better case detection—is placing increasing pressure on dermatology services, which must efficiently triage a rising number of pigmented and non-pigmented lesions.

Both melanoma and keratinocyte carcinoma develop through a well-established set of phenotypic and environmental risk factors, including fair skin phototype, high nevus count, family history of melanoma, and a history of sunburn—especially in childhood [5]. Because these risk factors are individually only weak-to-moderate predictors of malignancy, their translation into an actual clinical decision to biopsy or excise a lesion depends heavily on the treating clinician’s real-time morphological assessment rather than on anamnestic risk-factor counting alone.

Dermoscopy has become the standard tool used alongside naked-eye examination in this triage process. An insightful meta-analysis restricted to studies performed in genuine clinical settings found that dermoscopy raised the sensitivity of melanoma detection from 71% to 90% without a significant loss of specificity when compared with unaided visual inspection [6]. A subsequent systematic review focused specifically on primary care similarly concluded that structured dermoscopic triage algorithms improve referral accuracy and can reduce unnecessary excisions of benign lesions when appropriately implemented and audited [7]. This diagnostic gain underlies most contemporary referral and excision algorithms. However, the way in which dermoscopic impression actually translates into a surgical decision in everyday practice depends on a combination of demographic, historical, and morphological factors that has been less well studied using real-world screening data.

The clinical decision to excise a lesion carries direct consequences for both patient outcomes and health-system efficiency. Total-body skin examination programmes in dermatology practice have been reported to require a number needed to screen (NNS) of approximately 12 patients to detect one skin cancer of any type, and over 200 to detect one melanoma specifically [8]. A meta-analysis of the related number needed to treat/excise (NNT) metric across clinical settings found it ranged from roughly 23 in primary care to under 6 among pigmented lesion specialists [9], underscoring how strongly the pre-test risk profile of the screened population and the expertise of the assessing clinician influence screening yield.

Sex-related differences in health-seeking behaviour add another layer of complexity: survey-based studies consistently show that men are less likely than women to seek dermatological screening, more often present without a specific lesion of concern only when prompted by symptoms, and are under-represented among those who self-refer for total-body skin examination [10,11]. This pattern may paradoxically result in a higher surgical intervention rate once men do present for evaluation, if their pre-test risk profile at presentation is, on average, higher than that of women who seek screening earlier or more frequently.

Despite this large body of evidence on individual risk factors, few studies have demonstrated how demographic profile (age, sex), anamnestic risk factors (family history of melanoma, personal history of skin cancer, childhood sunburn history), phenotypic markers (nevus count), and dermoscopic impression (clinically atypical lesions, suspicion of BCC/SCC) combine to predict a real-world positive surgical decision (PSD) within a single screening cohort, or how these variables cluster into clinically interpretable patient profiles. The aim of the present study was therefore to evaluate, in a retrospective cohort of 643 patients undergoing dermoscopic skin cancer screening: (i) the demographic and clinical correlates of a positive surgical decision; (ii) the independent multivariable predictors of PSD, clinical atypia, and BCC/SCC suspicion; (iii) the natural clustering of patients into risk profiles using exploratory multivariate methods; and (iv) the diagnostic and screening efficiency of the dermoscopic classification system used in routine practice.

## 2. Materials and Methods

### 2.1. Study Population and Design

This retrospective, single-centre, cross-sectional analysis included 643 consecutive patients who underwent dermoscopic skin cancer screening. The cohort consisted of 415 women (64.5%) and 228 men (35.5%), with a mean age of 51.3 ± 16.8 years (median 52 years; interquartile range 39–65; range 18–84 years). Age did not differ significantly between sexes (Mann–Whitney Z = 0.64, *p* = 0.50), nor did the distribution across pre-specified age strata (<30, 30–49, 50–69, and ≥70 years; χ^2^ = 1.86, df = 3, *p* = 0.60), indicating a demographically balanced comparison between the male and female subgroups.

Patients were screened between January 2025 and December 2025. Eligible patients were consecutive attendees of the dermoscopic skin cancer screening service who had a complete record of the study variables and a documented outcome (surgical referral or continued observation); patients were excluded if they were under 18 years of age or if their dermoscopic record was incomplete. Screening in this cohort was organised as follows: participants were seen as part of “Znamiona pod lupą” (“Moles Under the Magnifying Glass”), a free, capacity-limited skin cancer screening programme for residents of Wrocław, run by the Department of Dermato-Venereology of the Fourth Military Clinical Hospital and financed by the City of Wrocław; participants registered in advance for a scheduled appointment rather than presenting opportunistically during a visit prompted by another concern. As this is a general population screening programme rather than a lesion-triggered consultation, most participants attended for a routine skin check rather than because of one specific lesion of concern, although individual participants could and did draw attention to a particular lesion during the intake interview.

Dermoscopic examinations were performed by board-certified dermatologists with formal training in dermoscopy. Examinations followed the ABCD rule of dermoscopy. The decision to recommend surgical excision was based on dermoscopic features consistent with malignancy or high-grade atypia. Surgical decisions were made by a single examining clinician.

### 2.2. Clinical Variables

The following anamnestic and clinical variables were abstracted for each patient: family history of melanoma (FHM), personal history of skin cancer (HSC), childhood sunburn (CS), clinically atypical lesions on dermoscopic examination (CAL), clinical suspicion of basal cell carcinoma or squamous cell carcinoma (BCC), nevus count coded on an ordinal 0–4 scale (NC), and the primary outcome of interest—a positive surgical decision (PSD), i.e., referral for excision of the index lesion. All binary variables were coded as present (1) versus absent (0).

Free-text entries in the dermoscopic description field were independently reviewed and categorised into one of seven pre-defined diagnostic classes: (1) no concern (e.g., “within normal limits”, “nevi without atypical features”, “typical age-related nevi”); (2) atypical nevus (e.g., “atypical pigmented nevus”, “pattern atypia”, “dysplastic nevus”, “network asymmetry”); (3) suspicion of BCC/cutaneous cancer (e.g., “suspected BCC”, “BCC for excision”, “erythematous lesion with telangiectasia”, “suspected melanoma”); (4) benign lesions (e.g., cherry angioma, fibroma, seborrhoeic keratosis, epidermal cyst, viral wart); (5) actinic keratosis (AK); (6) vascular lesions (e.g., cavernous haemangioma, venous lake); and (7) other pathology (e.g., psoriasis, acne, morphea, keratoacanthoma). Because some patients had more than one dermoscopic indication recorded, category frequencies do not sum to 100%.

### 2.3. Missing Data

Nevus count (NC) had the highest proportion of missing values among the study variables, with 85 of 643 records (13.2%) unrecorded (11.8% among women and 15.8% among men). To evaluate the missingness mechanism, Little’s test for data missing completely at random (MCAR) was applied using the naniar package (mcar_test function) (version 1.1.0) in R (version 4.5.3) (R Foundation for Statistical Computing, Vienna, Austria) [12]. The test yielded χ^2^ = 2.80, df = 2, *p* = 0.247 (two missing-data patterns), providing no evidence against the null hypothesis of MCAR and supporting the use of complete-case analysis for models involving NC.

### 2.4. Statistical Analysis

Continuous variables were tested for normality with the Shapiro–Wilk test; because age was not normally distributed, between-group comparisons used the Mann–Whitney U test rather than the *t*-test. Categorical variables were compared using the χ^2^ test or Fisher’s exact test where expected cell counts were small. Bivariate associations among binary and ordinal variables were quantified using Spearman’s rank correlation (*r*_s_) and the phi/Cramér’s V coefficient, respectively.

Multiple logistic regression models were built for three binary outcomes—PSD, CAL, and BCC/SCC suspicion—using demographic and clinical variables as candidate predictors, including a childhood sunburn × nevus count and an age × sex interaction term where clinically plausible. Full models were reduced by stepwise selection; model fit was assessed with the Hosmer–Lemeshow goodness-of-fit test and Nagelkerke pseudo-R^2^, and model parsimony with the Akaike and Bayesian information criteria (AIC, BIC).

To explore the joint structure of categorical clinical variables without imposing an a priori outcome, multiple correspondence analysis (MCA) was performed, following established recommendations for the use of correspondence analysis in epidemiological data. Patient-level clustering on the resulting mixed-type variable set (nominal, ordinal, and binary) was performed using partitioning-around-medoids (k-medoids/PAM) applied to a Gower dissimilarity matrix, computed with the daisy function of the R cluster package; this combination is well suited to mixed clinical data because it avoids the restrictive distributional assumptions of Euclidean distance methods.

Receiver operating characteristic (ROC) curve analysis was used to quantify the discriminative performance (area under the curve (AUC)), sensitivity, specificity, positive predictive value (PPV), and negative predictive value (NPV) of individual demographic and dermoscopic variables for PSD (pROC version 1.18.5). Referral efficiency was expressed as the number of patients screened per surgical referral (NSpSR), calculated as the reciprocal of the referral rate (NSpSR = 1/R_r_, where R_r_ is the proportion of screened patients referred for excision). Because this measure reflects the clinical decision to refer for excision rather than histologically confirmed cancer detection, it is conceptually distinct from the number needed to screen (NNS) or number needed to excise/treat (NNT) reported in the cancer screening literature and should not be compared directly with those figures. All tests were two-sided, with statistical significance set at *p* < 0.05.

## 3. Results

### 3.1. Demographic Characteristics of the Cohort

The demographic composition of the cohort, stratified by sex, is summarised in Table 1. Women predominated (64.5%) and the age distribution was closely comparable between sexes across all pre-specified strata.

### 3.2. Sex-Based Comparisons of Risk Factors and Surgical Decisions

Prevalence of anamnestic and clinical risk factors, with 95% confidence intervals, is presented in Table 2. Among all variables considered, only the surgical decision (PSD) differed significantly by sex: men were referred for excision significantly more often than women (25.9% vs. 17.9%; χ^2^ test, *p* = 0.017), corresponding to an odds ratio of approximately 1.61 (i.e., a 61% higher odds of a positive surgical decision in men).

### 3.3. Nevus Count and Data Completeness

Nevus count (NC, ordinal 0–4) did not differ significantly by sex (χ^2^ = 5.51, df = 5, *p* = 0.36; Table 3). NC had the largest proportion of missing data of all study variables (13.2% overall), which Little’s MCAR test (χ^2^ = 2.80, df = 2, *p* = 0.247) indicated was consistent with a completely random missingness mechanism, supporting complete-case handling in subsequent regression models.

### 3.4. Bivariate Associations with Surgical Decision and Clinical Atypia

Table 4 compares risk-factor prevalence between patients with and without a positive surgical decision. Suspicion of BCC/SCC was present in 19.7% of the PSD-positive group versus 1.0% of the PSD-negative group (*p* < 0.001), and clinically atypical lesions were present in 37.6% versus 9.8% (*p* < 0.001); women were under-represented in the PSD-positive group (55.6% vs. 66.8% female, *p* = 0.017), mirroring the sex-based comparison in Section 3.2. Family history, personal history, and childhood sunburn did not differ significantly between groups.

Table 5 presents the converse comparison, stratified by presence of clinically atypical lesions. Patients with CAL were referred for surgery in 50.0% of cases versus 15.3% of patients without CAL (*p* < 0.001), and reported childhood sunburn significantly more often (45.0% vs. 32.6%, *p* = 0.016), consistent with a phenotype–environment interaction explored further in Section 3.6.

### 3.5. Correlation Structure Among Clinical Variables

Spearman rank correlations among the nine study variables (Table 6) confirmed that the surgical decision correlated most strongly with suspicion of BCC/SCC (*r*_s_ = 0.353) and clinical atypia (*r*_s_ = 0.310), while correlations with family history, personal history, and childhood sunburn were weak (|*r*_s_| ≤ 0.065). Age correlated modestly with both personal history of skin cancer (*r*_s_ = 0.147) and suspicion of BCC/SCC (*r*_s_ = 0.176), consistent with an age-related increase in non-melanoma skin cancer risk. Nevus count correlated with family history of melanoma (*r*_s_ = 0.167), suggesting a partly heritable basis for nevus phenotype.

### 3.6. Multivariable Logistic Regression Models

Univariable and multivariable logistic regression results for the three outcomes of interest (PSD, CAL, and BCC/SCC suspicion) are presented in Table 7, Table 8 and Table 9.

For PSD (Table 7), univariable analysis identified suspicion of BCC/SCC (OR = 24.7, 95% CI 9.26–66.0), clinical atypia (OR = 5.53, 95% CI 3.50–8.74), age (OR = 1.02 per year, *p* = 0.008), and female sex (OR = 0.62, *p* = 0.017) as significant predictors. In the final multivariable model, only sex, CAL, and BCC/SCC suspicion remained independent predictors:

Logit *P*r{PSD = 1} = −2.298 − 0.692 × (Female) + 1.931 × (CAL) + 3.616 × (BCC)

This model showed excellent calibration (Hosmer–Lemeshow χ^2^ = 2.12, df = 8, *p* = 0.977) and explained 31% of outcome variance (Nagelkerke *R*^2^ = 0.31; AIC = 533.33; BIC = 573.52; df = 639).

For CAL (Table 8), univariable analysis identified childhood sunburn (OR = 1.69), nevus count (OR = 1.65 per category), PSD (OR = 5.53), and a significant CS × NC interaction (OR = 1.39, 95% CI 1.16–1.66, *p* < 0.001) as predictors. The final reduced model retained only nevus count and PSD:

Logit *P*{CAL = 1} = −3.176 + 0.507 × (NC) + 1.714 × (PSD)

Model fit was weaker than for PSD (Hosmer–Lemeshow χ^2^ = 13.6, df = 8, *p* = 0.018; Nagelkerke *R*^2^ = 0.19; AIC = 486.11; BIC = 499.51; df = 639), suggesting residual unexplained variability in the clinical determination of atypia.

For suspicion of BCC/SCC (Table 9), age (OR = 1.06 per year, *p* < 0.001) and PSD (OR = 24.7) were significant univariable predictors, while sex, family/personal history, sunburn, nevus count, and CAL were not. The multivariable model reduced to a single predictor:

Logit *P*{BCC = 1} = −4.613 + 3.208 × (PSD)

indicating that, within this cohort, a clinical suspicion of BCC/SCC was almost synonymous with the decision to excise (Hosmer–Lemeshow χ^2^ = 21.4, df = 8, *p* = 0.006; Nagelkerke R^2^ = 0.37; AIC = 174.82; BIC = 192.66; df = 634).

Patients with clinically suspected BCC/SCC (*n* = 31) were significantly older (mean 64.1 ± 10.6 vs. 50.7 ± 16.9 years; Mann–Whitney Z = 4.45, *p* < 0.001) than the remainder of the cohort (*n* = 611–612), with none aged under 30 years, and were referred for surgery far more often (83.9% vs. 17.3%; *p* < 0.001). Sex distribution, family history, personal history, sunburn history, and clinical atypia did not differ significantly between the BCC/SCC-suspicion group and the rest of the cohort (all *p* > 0.20). Age alone discriminated the BCC/SCC-suspicion group reasonably well (AUC = 0.737, 95% CI 0.667–0.807), with a cut-off of ≥60 years yielding a sensitivity of 77.4%, specificity of 67.5%, and accuracy of 67.9%.

### 3.7. Dermoscopic Classification and Its Association with Surgical Decision

Categorisation of free-text dermoscopic descriptions yielded seven diagnostic classes (Table 10). Almost two-thirds of patients (64.1%) had no dermoscopic concern recorded, while atypical nevi and suspicion of BCC accounted for 18.2% and 2.8% of records, respectively. Because 4.8% of patients had more than one indication recorded (4.2% two indications, 0.6% three), category percentages exceed the number of unique patients.

Table 11 presents three logistic regression models predicting the surgical decision from sex, age, and each dermoscopic category in turn. Absence of concern was strongly protective against excision (OR = 0.10, 95% CI 0.06–0.16), atypical lesions carried a markedly elevated odds of excision (OR = 19.1, 95% CI 11.3–32.2), and suspicion of BCC carried the highest odds (OR = 21.3, 95% CI 5.96–75.9). Female sex remained independently protective (OR 0.57–0.62) and age was a positive, though modest, predictor in the models including atypia or BCC suspicion.

### 3.8. Exploratory Multivariate Analysis: Correspondence Analysis and Cluster Profiles

Multiple correspondence analysis (MCA) was performed across all categorical clinical variables. The first two dimensions explained 11.5% and 9.63% of total inertia, respectively (Figure S1). The first dimension separated a high-risk profile—characterised by joint proximity of CAL:yes, BCC:yes, and PSD:yes (dimension-1 coordinates 1.426, 2.289, and 1.455, respectively)—from the majority “observed” population (CAL:no, PSD:no, BCC:no, NC:0). PSD:yes contributed most strongly to the structure of the map (cos^2^ = 0.547), consistent with the logistic regression findings in Section 3.6, while HSC:yes was distinct along the second dimension, indicating a separate profile of patients with a personal oncological history.

A Gower dissimilarity matrix computed across mixed-type variables (age group, sex, FHM, HSC, CS, NC, CAL, BCC, PSD) was clustered using k-medoids (PAM), yielding five clinically distinguishable patient profiles (Table S1, Figure S1), visualised by two-dimensional multidimensional scaling. The first MDS axis separated clusters primarily by clinical severity (nevus burden and surgical intervention), while the second axis reflected within-cluster heterogeneity in clusters 4 and 5.

### 3.9. Diagnostic Performance and Screening Efficiency

A composite risk score combining demographic and clinical risk factors discriminated PSD with an AUC of 0.783, indicating good overall discriminatory ability. When individual variables were evaluated separately (Table 12), “no concern” was the single best predictor (AUC = 0.762) with the highest negative predictive value (NPV = 0.927), meaning that a dermoscopic impression of “no concern” effectively ruled out surgical intervention. “Atypical lesion” combined high specificity (0.916) with the highest positive predictive value among common categories (PPV = 0.632; AUC = 0.736). Rarer categories (suspicion of BCC, actinic keratosis, vascular lesions) showed very high specificity (>0.98) but low sensitivity, reflecting their low prevalence. Age and sex alone were weak discriminators of PSD (AUC 0.556–0.574).

Across the full cohort, 133 of 643 screened patients (20.7%) were referred for excision, giving a referral rate of *R_r_* = 0.207 and a number of patients screened per surgical referral of NSpSR = 1/0.207 ≈ 4.8—that is, approximately five patients needed to be examined to generate one surgical referral. This figure describes referral efficiency within the present dermoscopy-led triage pathway and does not represent the number needed to screen to detect one histologically confirmed skin cancer.

## 4. Discussion

In this retrospective cohort of 643 patients undergoing dermoscopic skin cancer screening, the decision to proceed to surgical excision was overwhelmingly driven by the immediate dermoscopic impression rather than by anamnestic oncological risk factors. Suspicion of BCC/SCC increased the odds of excision approximately 21- to 25-fold across models, and clinical atypia increased it approximately 5.5- to 19-fold, whereas family history of melanoma and personal history of skin cancer—both classical anamnestic risk markers—were not independently associated with the surgical decision in any model. This pattern is clinically reassuring; it suggests that, in this practice setting, excision decisions were guided by the morphological features most directly linked to malignancy risk, rather than by patient anxiety or a general tally of risk factors. This is consistent with the reasoning behind dermoscopy-based triage algorithms more broadly [6,7]. The strong age-dependence of BCC/SCC suspicion observed in this cohort (mean age 64.1 vs. 50.7 years; age alone AUC = 0.737) is also consistent with the cumulative, latency-dependent relationship between lifetime ultraviolet exposure and basal cell carcinoma risk described in recent reviews of BCC pathogenesis [13].

From a bedside decision-making perspective, PPV and NPV are arguably more directly useful to the practising dermatologist than AUC alone, because—unlike AUC, which summarises discrimination across every possible threshold—they describe the operating characteristics of the specific dermoscopic impression actually used in routine practice [14]. In this cohort, a dermoscopic impression of “no concern” carried an NPV of 0.927, meaning that fewer than 1 in 13 patients given this reassuring impression ultimately had a positive surgical decision, supporting safe deferral of excision in this group. Conversely, “atypical lesion” carried a PPV of 0.632 together with a specificity of 0.916, meaning that roughly two in three patients assigned this category were referred for surgery and that this impression can be used with reasonable confidence to justify referral. We therefore emphasise PPV and NPV, rather than AUC alone, as the metrics of greatest direct relevance to bedside triage decisions in this setting.

Beyond dermoscopic triage itself, once a lesion is flagged as suspicious for BCC/SCC and referred for excision, imaging modalities that estimate tumour depth and extent pre-operatively can further inform surgical planning. Recent work using a portable 20 MHz high-frequency ultrasound (HFUS) device has shown good agreement between ultrasound-estimated and histopathologically confirmed basal cell carcinoma depth, including a depth-correction model that improves pre-operative estimation accuracy [15]. Incorporating point-of-care HFUS assessment after dermoscopic triage identifies a suspicious lesion, but before the surgical decision is finalised, could complement the dermoscopic impression captured in the present study by providing an objective, pre-operative estimate of tumour extent—particularly for BCC, where depth is not reliably inferred from surface dermoscopic features alone. Combining dermoscopic and sonographic triage represents a promising avenue for future prospective work.

A consistent and independent finding across every model was that women had significantly lower odds of a positive surgical decision than men (OR 0.57–0.62 across models; unadjusted 17.9% vs. 25.9%, *p* = 0.017). This is broadly congruent with survey evidence that men are less likely than women to seek dermatological screening proactively and tend to present with more advanced or clinically concerning lesions when they eventually do seek care [10,11]. It is also in line with—though not directly comparable to—population-based cohort evidence showing that male sex is independently associated with more advanced disease stage at diagnosis, along with a 20–50% higher risk of melanoma recurrence and melanoma-specific mortality [16]. It also aligns with registry-based data showing higher age-specific melanoma incidence among men over 50 years, together with a markedly different anatomical distribution of tumours between the sexes [17]. If men in this cohort were, on average, presenting later or with a higher pre-test probability of a truly concerning lesion, a higher observed excision rate among men would be an expected downstream consequence of differential health-seeking behaviour rather than evidence of sex-based bias in clinical decision-making per se. Because the present dataset did not capture time since symptom onset or screening-seeking motivation, this explanation cannot be directly tested here and should be examined in future prospective work that links anamnestic screening behaviour to dermoscopic and histopathological outcomes.

The significant childhood sunburn × nevus count interaction identified for clinical atypia (OR = 1.39, 95% CI 1.16–1.66, *p* < 0.001) reinforces a dual environmental–phenotypic pathway to atypical nevus development that is well documented in the paediatric and adolescent nevus literature: sunburn history and cumulative sun exposure in childhood are established correlates of higher adult nevus counts and of atypical nevus phenotype, and their joint effect on nevus accumulation appears synergistic rather than simply additive [18]. In the present adult screening cohort, this translated into a measurable interaction effect on clinically apparent atypia, supporting the biological plausibility of combining sunburn history and nevus count—rather than either alone—when stratifying patients for closer dermoscopic surveillance.

The referral efficiency observed in this cohort—approximately one surgical referral generated for every 4.8 patients screened (NSpSR ≈ 4.8)—reflects the yield of the present dermoscopy-led triage pathway rather than a measure of cancer-detection efficiency per se. Because PSD captures a broader, clinician-defined category of lesions considered to warrant excision—including some that were not ultimately histologically confirmed as malignant—this figure is not directly comparable to published estimates of the number needed to screen (NNS) or number needed to excise/treat (NNT), which are anchored to histologically confirmed cancer detection, such as the NNS of approximately 12 reported for total-body skin examination in general dermatology practice [8] or NNT estimates ranging from roughly 6 among pigmented-lesion specialists to over 20 in primary care [9]. A recent Swedish cohort study illustrates, in a qualitative sense, how strongly triage infrastructure can shift such metrics: introducing teledermoscopy-supported triage nearly halved the number needed to excise in the same population (from 23.3 to 9.0) while significantly reducing Breslow thickness at diagnosis—underscoring that NNS/NNT-type values are only meaningful within the specific referral pathway, population, and endpoint definition used, and cannot be used to rank screening programmes against one another without a shared, histologically confirmed endpoint [19]. A methodologically sound comparison would require linking PSD outcomes in this cohort to histopathology reports so that a true, histologically anchored NNS/NNT could be calculated; this is an important direction for prospective follow-up work. What the present referral efficiency figure does indicate is that, within this triage pathway, dermoscopic impression concentrated surgical referrals in a relatively small, high-yield subset of the screened population, consistent with the discriminative performance reported in Section 3.9.

The exploratory multiple correspondence analysis and Gower-distance k-medoids clustering (Section 3.8; Table S1, Figure S1) resolved the cohort into five interpretable clinical profiles, ranging from a low-risk observational group to a very-high-risk group with the highest nevus burden, atypia, and surgical intervention rate. Because PSD:yes contributed most strongly to the first MCA dimension (cos^2^ = 0.547), this was internally consistent with—rather than independent evidence beyond—the regression-based findings above and should be read as a descriptive typology for clinical communication and hypothesis generation rather than a validated risk-stratification tool.

Missing nevus-count data were formally evaluated as MCAR rather than assumed, supporting complete-case analysis; the exploratory correspondence analysis/clustering results (Section 3.8) provide a complementary, hypothesis-generating view of the same associations identified by regression (see Discussion above). Several limitations should be considered when interpreting these findings. First, the study was retrospective, single-centre, and cross-sectional, precluding causal inference and limiting generalisability to other populations and referral pathways. Second, the primary outcome (PSD) was a clinical decision to excise rather than a histopathologically confirmed diagnosis; consequently, the reported associations describe determinants of clinician decision-making rather than determinants of malignancy per se, and some excised lesions classified as “suspicion of BCC/SCC” or “atypical” may not have been histologically confirmed as malignant or dysplastic—a distinction that matters in light of broader population-level concerns that intensified screening can lead to the detection and excision of clinically indolent disease that would not otherwise have become apparent [4]. Third, the subgroup with confirmed suspicion of BCC/SCC was small (*n* = 31), and the corresponding regression model, while statistically significant, should be interpreted cautiously given the wide confidence intervals around its odds ratios and the modest Hosmer–Lemeshow fit (*p* = 0.006). Fourth, categorisation of free-text dermoscopic descriptions into seven diagnostic classes, although performed against pre-specified criteria, necessarily involved a degree of interpretive judgement and could not be independently validated against dermoscopic images in this analysis. Fifth, dermoscopic suspicion of keratinocyte carcinoma was recorded as a single composite “BCC/SCC” category reflecting real-world triage documentation rather than as separate basal cell carcinoma and squamous cell carcinoma entities; because BCC is generally diagnosed with higher dermoscopic accuracy than SCC, pooling the two may have obscured differences in referral performance between them, and this composite variable could not be disaggregated within the present dataset [20]. Future studies should record and analyse BCC and SCC suspicion as separate diagnostic categories, ideally with histopathological subtype confirmation. Sixth, because dermoscopic diagnostic accuracy and inter-rater agreement are known to be strongly experience-dependent, the referral efficiency and diagnostic-performance figures reported here may not generalise to settings with less experienced examiners, and no formal inter-rater reliability analysis was performed in this retrospective, single-reader-per-case design [21]. Seventh, as with any observational dataset compiled from clinical records, occasional internal inconsistencies were identified during data verification (Section 3.4); these were corrected against the underlying counts where possible, but their presence highlights the importance of source-data verification in retrospective registry-based research.

## 5. Conclusions

In this cohort of 643 patients undergoing dermoscopic skin cancer screening, the decision to proceed with surgical excision was driven almost entirely by the dermoscopic impression—particularly suspicion of BCC/SCC and clinical atypia—rather than by family or personal oncological history. Male sex was independently associated with a higher surgical rate, a pattern that may reflect differences in screening-seeking behaviour rather than clinical bias. A simple, two-category dermoscopic classification (“no concern” versus “atypical/suspicious”) captured most of the information relevant to the surgical decision: “no concern” showed a high negative predictive value (0.927), supporting safe deferral of intervention, while “atypical lesion” showed high specificity and positive predictive value (0.916 and 0.632, respectively), supporting referral for excision. The dermoscopic classification also generated a favourable referral efficiency figure in this cohort (NSpSR ≈ 5, i.e., approximately one surgical referral for every five patients screened); however, because this reflects a clinical referral decision rather than histologically confirmed cancer detection, it is best interpreted as a measure of triage yield within this specific pathway rather than as directly comparable to published cancer-detection screening benchmarks, and this likely reflects the pre-selected nature of the screened population rather than the screening technique itself. These findings support the continued use of dermoscopy-led triage in routine skin cancer screening and suggest that combining childhood sunburn history with nevus count could help identify patients at risk of clinical atypia. Prospective, multicentre studies incorporating histopathological confirmation are needed to validate these decision-support patterns before clinical implementation.

## Figures and Tables

**Table 1:** Demographic characteristics of the study cohort, overall and by sex.

Variable	Total *N* = 643 (100%)	Female *N* = 415 (64.5%)	Male *N* = 228 (35.5%)	Test Statistic
Age (years)				
Mean ± SD	51.3 ± 16.8	51.8 ± 16.4	50.4 ± 17.7	Z = 0.64
Median (Q1;Q3)	52 (39;65)	52 (39;66)	52 (39;65)	*p* = 0.50
Min–Max	18–84	18–84	18–84	
Age group				
<30 years	80 (12.4%)	47 (11.3%)	33 (14.5%)	χ^2^ = 1.86, df = 3
30–49 years	206 (32.0%)	138 (33.3%)	68 (29.8%)	*p* = 0.60
50–69 years	240 (37.3%)	153 (36.9%)	87 (38.2%)	
≥70 years	117 (18.2%)	77 (18.5%)	40 (17.5%)	

**Table 2:** Clinical risk factors and surgical decision, overall and by sex (*n*, % (95% CI)).

Variable	Total *N* = 643	Female *N* = 415	Male *N* = 228	*p*
Family history of melanoma (FHM)	40 (6.3%) (4.4–8.1)	28 (6.8%) (4.3–9.2)	12 (5.3%) (2.4–8.2)	0.443
Personal history of skin cancer (HSC)	17 (2.6%) (1.4–3.9)	9 (2.2%) (0.8–3.6)	8 (3.5%) (1.1–5.95)	0.311
Childhood sunburn (CS)	222 (34.5%) (30.9–38.2)	154 (37.1%) (32.5–41.8)	68 (29.8%) (23.9–35.8)	0.063
Clinically atypical lesions (CAL)	100 (15.6%) (12.8–18.4)	66 (15.9%) (12.4–19.4)	34 (14.9%)(10.3–19.5)	0.740
Suspicion of BCC/SCC (BCC)	31 (4.8%) (3.2–6.5)	21 (5.1%) (3.0–7.2)	10 (4.4%) (1.7–7.0)	0.698
**Positive surgical decision (PSD)**	**133 (20.7%) (** **17.6–23.8** **)**	**74 (17.9%) (** **14.1–21.5** **)**	**59 (25.9%) (** **20.2–31.6** **)**	**0.017**

95% CI: 95% confidence interval for the proportion. Bold rows denote the statistically significant sex comparison (OR ≈ 1.61 for men vs. women).

**Table 3:** Distribution of nevus count (NC), overall and by sex.

NC Category	Total *N* = 643	Female *N* = 415	Male *N* = 228	Test Statistic
0	20 (3.1%)	14 (3.4%)	6 (2.6%)	χ^2^ = 5.51, df = 5
1	196 (30.5%)	126 (30.4%)	70 (30.7%)	*p* = 0.36
2	206 (32.0%)	129 (31.1%)	77 (33.8%)	
3	93 (14.5%)	68 (16.4%)	25 (11.0%)	
4	43 (6.7%)	29 (7.0%)	14 (6.1%)	
Missing	85 (13.2%)	49 (11.8%)	36 (15.8%)	

**Table 4:** Bivariate comparison of risk factors by surgical decision (PSD).

Variable, *n* (%)	PSD Positive (*n* = 133)	PSD Negative (*n* = 509–510)	*p*
Female sex	74/133 (55.6%)	340/509 (66.8%)	0.017
Family history of melanoma (FHM)	5/132 (3.8%)	35/507 (6.9%)	0.188
Personal history of skin cancer (HSC)	3/133 (2.3%)	14/509 (2.8%)	0.752
Childhood sunburn (CS)	54/133 (40.6%)	168/509 (33.0%)	0.101
**Clinically atypical lesions (CAL)**	**50/133 (37.6%)**	**50/509 (9.8%)**	**<0.001**
**Suspicion of BCC/SCC**	**26/132 (19.7%)**	**5/509 (1.0%)**	**<0.001**

Bold text indicates statistical significance (*p* < 0.05). BCC/SCC: basal cell carcinoma or squamous cell carcinoma; PSD positive: outcome variable present (patient referred for surgical excision); PSD negative: outcome variable absent (continued observation, no excision).

**Table 5:** Bivariate comparison of risk factors and outcomes by presence of clinically atypical lesions (CAL).

Variable, *n* (%)	CAL Present (*n* = 99–100)	CAL Absent (*n* = 541–543)	*p*
Female sex	66/100 (66.0%)	349/543 (64.3%)	0.740
Family history of melanoma (FHM)	5/99 (5.1%)	35/541 (6.5%)	0.592
Personal history of skin cancer (HSC)	3/100 (3.0%)	14/543 (2.6%)	0.809
**Childhood sunburn (CS)**	**45/100 (45.0%)**	**177/543 (32.6%)**	**0.016**
Suspicion of BCC/SCC	4/99 (4.0%)	27/543 (5.0%)	0.691
**Positive surgical decision (PSD)**	**50/100 (50.0%)**	**83/542 (15.3%)**	**<0.001**

Bold text indicates statistical significance (*p* < 0.05). BCC/SCC: basal cell carcinoma or squamous cell carcinoma; CAL: clinically atypical lesions;

**Table 6:** Spearman correlation matrix among study variables (lower triangular).

Variable	Age	Female	FHM	HSC	CS	NC	CAL	BCC	PSD
Female	−0.027	×							
FHM	0.014	−0.030	×						
HSC	0.147 ***	0.040	−0.003	×					
CS	0.015	−0.073	0.083 *	−0.059	×				
NC	−0.042	−0.036	0.167 ***	0.036	0.021	×			
CAL	−0.006	−0.013	−0.021	0.010	0.095 *	0.153 ***	×		
BCC/SCC	0.176 ***	−0.015	0.062	0.053	0.005	0.003	−0.016	×	
PSD	0.105 **	0.094 *	−0.052	−0.012	0.065	0.030	0.310 ***	0.353 ***	×

* *p* < 0.05; ** *p* < 0.01; *** *p* < 0.001. FHM: family history of melanoma; HSC: personal history of skin cancer; CS: childhood sunburn; NC: nevus count; CAL: clinically atypical lesions; BCC/SCC: suspicion of BCC/SCC; PSD: positive surgical decision. The × symbol denotes the diagonal of the matrix where variables correlate with themselves. The “×” symbol denotes the diagonal of the matrix where variables correlate with themselves.

**Table 7:** Univariable odds ratios (OR) and 95% CI for a positive surgical decision (PSD).

Variable	OR	2.5% CI	97.5% CI	*p*
(Intercept)	0.35	0.26	0.47	<0.001
Age (years)	1.02	1.00	1.03	0.008
Female sex	0.62	0.42	0.92	0.017
Family history (FHM)	0.53	0.20	1.39	0.195
Personal history (HSC)	0.82	0.23	2.89	0.752
Childhood sunburn (CS)	1.39	0.94	2.06	0.102
Nevus count (NC)	1.15	0.97	1.37	0.106
Clinically atypical lesions (CAL)	5.53	3.50	8.74	<0.001
Suspicion of (BCC/SCC)	24.7	9.26	66.0	<0.001

OR: odds ratio; CI: confidence interval; BCC/SCC: basal cell carcinoma or squamous cell carcinoma;.

**Table 8:** Univariable odds ratios (OR) and 95% CI for clinically atypical lesions (CAL), including the CS × NC interaction.

Variable	OR	2.5% CI	97.5% CI	*p*
(Intercept)	0.18	0.12	0.25	<0.001
Age (years)	1.00	0.99	1.01	0.892
Female sex	1.08	0.70	1.69	0.739
Family history (FHM)	0.77	0.29	2.02	0.593
Personal history (HSC)	1.17	0.33	4.15	0.809
Childhood sunburn (CS)	1.69	1.10	2.61	0.018
Nevus count (NC)	1.65	1.35	2.02	<0.001
Suspicion of BCC/SCC	0.80	0.27	2.36	0.691
Positive surgical decision (PSD)	5.53	3.50	8.74	<0.001
Age × Female	1.00	0.99	1.01	0.549
CS × NC	1.39	1.16	1.66	<0.001

OR: odds ratio; CI: confidence interval; FHM: family history of melanoma; HSC: personal history of skin cancer; CS: childhood sunburn; NC: nevus count; BCC/SCC: basal cell carcinoma or squamous cell carcinoma; PSD: positive surgical decision.

**Table 9:** Univariable odds ratios (OR) and 95% CI for suspicion of BCC/SCC.

Variable	OR	2.5% CI	97.5% CI	*p*
(Intercept)	0.05	0.04	0.08	<0.001
Age (years)	1.06	1.03	1.09	<0.001
Female sex	1.16	0.54	2.53	0.699
Family history (FHM)	2.35	0.78	7.11	0.129
Personal history (HSC)	2.74	0.60	12.6	0.195
Childhood sunburn (CS)	1.05	0.49	2.24	0.899
Nevus count (NC)	1.05	0.76	1.47	0.743
Clinically atypical lesions (CAL)	0.80	0.27	2.36	0.691
Positive surgical decision (PSD)	24.7	9.28	65.9	<0.001

CI: confidence interval.

**Table 10:** Categorisation of dermoscopic findings.

Dermoscopic Category	*N* (%)	Illustrative Descriptor Examples
1. No concern	412 (64.1%)	“Within normal limits”; typical age-related nevi
2. Atypical nevus	116 (18.2%)	Pattern atypia; network asymmetry
3. Suspicion of BCC/skin cancer	18 (2.8%)	Erythematous lesion with telangiectasia; suspected BCC
4. Benign lesions	94 (14.6%)	Cherry angioma; fibroma; seborrhoeic keratosis
5. Actinic keratosis (AK)	19 (3.0%)	Solar keratosis; sun-damage foci
6. Vascular lesions	10 (1.6%)	Cavernous haemangioma; venous lake
7. Other pathology	8 (1.2%)	Psoriasis; morphea; keratoacanthoma
“For excision” annotation	28 (4.4%)	

Percentages do not sum to 100% because some patients had more than one dermoscopic indication (95.2% one indication, 4.2% two, 0.6% three). BCC: Basal cell carcinoma.

**Table 11:** Logistic regression models predicting the surgical decision from sex, age, and dermoscopic category.

Model/Predictor	*b*	*p*	OR (95% CI)
Model A: “No concern”			
Intercept	−0.441	0.273	0.64 (0.29; 1.42)
Female sex	−0.475	0.036	0.62 (0.40; 0.96)
Age (years)	0.009	0.155	1.01 (0.99; 1.02)
No concern (present)	−2.288	<0.001	0.10 (0.06; 0.16)
Model B: “Atypical lesion”			
Intercept	−3.722	<0.001	0.02 (0.01; 0.06)
Female sex	−0.532	0.024	0.59 (0.37; 0.93)
Age (years)	0.035	<0.001	1.04 (1.02; 1.06)
Atypical lesion (present)	2.949	<0.001	19.1 (11.3; 32.2)
Model C: “Suspicion of BCC”			
Intercept	−1.820	<0.001	0.16 (0.08; 0.32)
Female sex	−0.569	0.006	0.57 (0.38; 0.85)
Age (years)	0.014	0.027	1.01 (1.00; 1.03)
Suspicion of BCC (present)	3.057	<0.001	21.3 (5.96; 75.9)

OR: odds ratio; CI: confidence interval; BCC: Basal cell carcinoma.

**Table 12:** ROC-derived diagnostic performance of demographic and dermoscopic variables for the surgical decision (PSD).

Variable	AUC (95% CI)	Cut-Off	Sens.	Spec.	PPV	NPV
Age	0.574 (0.52; 0.63)	≥60 y	0.466	0.684	0.278	0.831
Female sex	0.556 (0.50; 0.61)	Yes	0.444	0.669	0.331	0.556
No concern	0.762 (0.72; 0.81)	Yes	0.774	0.749	0.446	0.927
Atypical lesion	0.736 (0.68; 0.79)	Yes	0.556	0.916	0.632	0.888
Suspicion of BCC	0.553 (0.50; 0.61)	Yes	0.113	0.994	0.833	0.811
Benign lesions	0.507 (0.45; 0.56)	No	0.865	0.149	0.209	0.809
Actinic keratosis (AK)	0.524 (0.47; 0.58)	Yes	0.068	0.980	0.792	0.474
Vascular lesions	0.514 (0.46; 0.57)	Yes	0.038	0.990	0.793	0.500
Other pathology	0.503 (0.45; 0.56)	No	0.992	0.014	0.208	0.875

CI: confidence interval; PPV: positive predictive value; NPV: Negative predictive value; BCC: Basal cell carcinoma.

## Data Availability

The data presented in this study are available on request from the corresponding author. The data are not publicly available owing to institutional data-sharing restrictions.

## Supplementary Materials

The following supporting information can be downloaded at https://www.mdpi.com/article/10.3390/medicina62091655/s1, Table S1: K-medoid (PAM, Gower distance) cluster profiles: category-coded centroid means and clinical interpretation; Figure S1: Multiple correspondence analysis (MCA) factor map.

## Abbreviations

The following abbreviations are used in this manuscript:

AIC/BIC	Akaike/Bayesian information criterion
AK	Actinic keratosis
AUC	Area under the receiver operating characteristic curve
BCC	Basal cell carcinoma
CAL	Clinically atypical lesion(s)
CI	Confidence interval
CS	Childhood sunburn
FHM	Family history of melanoma
HSC	Personal history of skin cancer
MCA	Multiple correspondence analysis
MCAR	Missing completely at random
NC	Nevus count
NNS	Number needed to screen (as used in the published literature comparisons only; the study’s own metric is NSpSR, below)
NSpSR	Number of patients screened per surgical referral
NPV/PPV	Negative/positive predictive value
OR	Odds ratio
PAM	Partitioning around medoids (k-medoids)
PSD	Positive surgical decision
ROC	Receiver operating characteristic
SCC	Squamous cell carcinoma
