# Supplementary material for: Clinical and Dermoscopic Determinants of Surgical Decision-Making in Skin Cancer Screening: A Retrospective Analysis
Source: Medicina (Kaunas). 2026 Aug 28;62(9):1655. doi: 10.3390/medicina62091655 (PMC13608977; doi:10.3390/medicina62091655)
Supplement: Supplementary file 1 [file medicina-62-01655-s001.zip › medicina-4470895-supplementary.pdf]

Table S1. K-medoids (PAM, Gower distance) cluster profiles: category-coded centroid means and clinical interpretation.

Cluster	Age	Female	FHM	HSC	CS	NC	CAL	BCC	PSD	Profile
1	2.58	2.00	1.10	1.01	2.00	2.79	1.14	1.05	1.13	High nevus burden, frequent CAL and PSD
2	2.70	2.00	1.05	1.03	1.00	2.63	1.04	1.05	1.07	Observational: moderate nevi, infrequent CAL
3	2.54	1.00	1.04	1.04	1.00	2.47	1.05	1.04	1.16	Low risk: few nevi, low CAL frequency
4	2.44	1.75	1.05	1.03	1.24	3.19	1.86	1.08	1.90	Very high risk: highest nevi, CAL and PSD
5	2.70	1.00	1.09	1.00	2.00	2.65	1.17	1.05	1.32	Atypia-risk: frequent CAL, lower PSD than cluster 4

Values are category-coded centroid means as produced by the clustering algorithm (e.g., 1 = reference/absent category, 2 = present category for binary variables), not raw probabilities; they are reported here exactly as generated for cluster differentiation, not as clinical proportions. FHM: Family history of melanoma; HSC: Personal history of skin cancer; CS: Childhood sunburn; NC: Nevus count; CAL: Clinically atypical lesion(s); BCC: Basal cell carcinoma; PSD: Positive surgical decision.

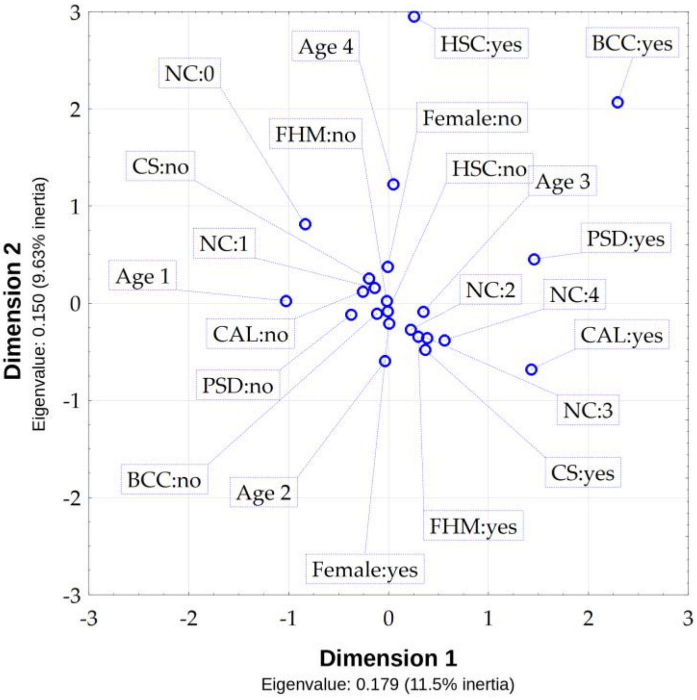

Figure S1. Multiple correspondence analysis (MCA) factor map (Dimension 1 vs. Dimension 2, explaining 11.5% and 9.63% of total inertia, respectively), with individual patients coloured by k-medoids (PAM, Gower-distance) cluster assignment (Clusters 1–5, as defined in Table 12). Category coordinates for clinically atypical lesions (CAL:yes), suspicion of BCC/SCC (BCC:yes), and

positive surgical decision (PSD:yes) cluster together along Dimension 1, consistent with their shared contribution to the high-risk patient profile, while personal history of skin cancer (HSC:yes) separates along Dimension 2.
